# Supplementary material for: A rationally engineered decoder of transient intracellular signals
Source: Nat Commun. 2021 Mar 25;12:1886. doi: 10.1038/s41467-021-22190-4 (PMC7994635; doi:10.1038/s41467-021-22190-4)
Supplement: Supplementary file 1 — Supplementary Information [file 41467_2021_22190_MOESM1_ESM.pdf]

Supplementary Information for
A rationally engineered decoder of transient intracellular signals

Claude Lormeau, Fabian Rudolf, Jörg Stelling
Correspondence to: joerg.stelling@bsse.ethz.ch

This PDF file includes:

Supplementary Figures 1 to 10

Supplementary Methods

Supplementary Tables 1 to 8

Supplementary Figures

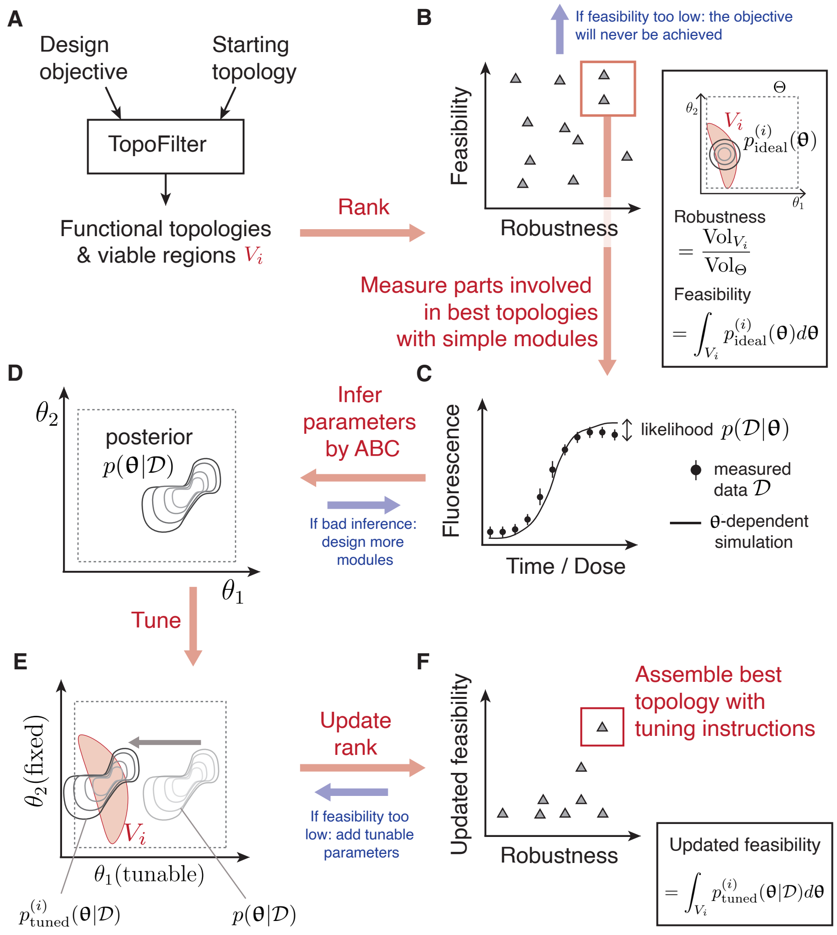

Supplementary Fig. 1 – Summary of the TopoDesign method. Red arrows indicate the successful flow of the method, blue arrows indicate potential exit routes or returns which can be done early enough, avoiding the need to build more than one complete circuit. **(A)** The design objective and starting topology are fed to the topological filtering algorithm, which finds viable topologies T_i obtained by removing interactions from the starting topology. The viable topologies achieve the design objective for at least one parameter set θ. For each topology T_i we also obtain the viable region V_i. **(B)** The feasibility based on an ideal Gaussian prior and the robustness of viable circuits are computed and we obtain a first ranking. We then observe the viable space of the best topologies to see if some constraints are recurrent. This step gives a first overview of possible topologies to achieve our goal, and helps selecting which parts will be assembled. **(C)** With the selected parts, simple modules are assembled and their dose response and/or dynamics are measured, leading to a calibration data set. A distance function is defined that compares the calibration data to the simulation of the modules by mathematical models. **(D)** By approximate Bayesian computation we infer the posterior probability of parameters given our calibration data set. **(E)** The location of the posterior is shifted in the direction of tunable parameters, to maximize the updated feasibility, which is now based on the inferred posterior instead of an ideal Gaussian prior. **(F)** The updated ranking of topologies should enable to discriminate which topology is more likely to achieve the design objective in the context of our lab. The magnitude of the shifts in tunable directions operated in step (E) provides guidance for the assembly of the best circuits.

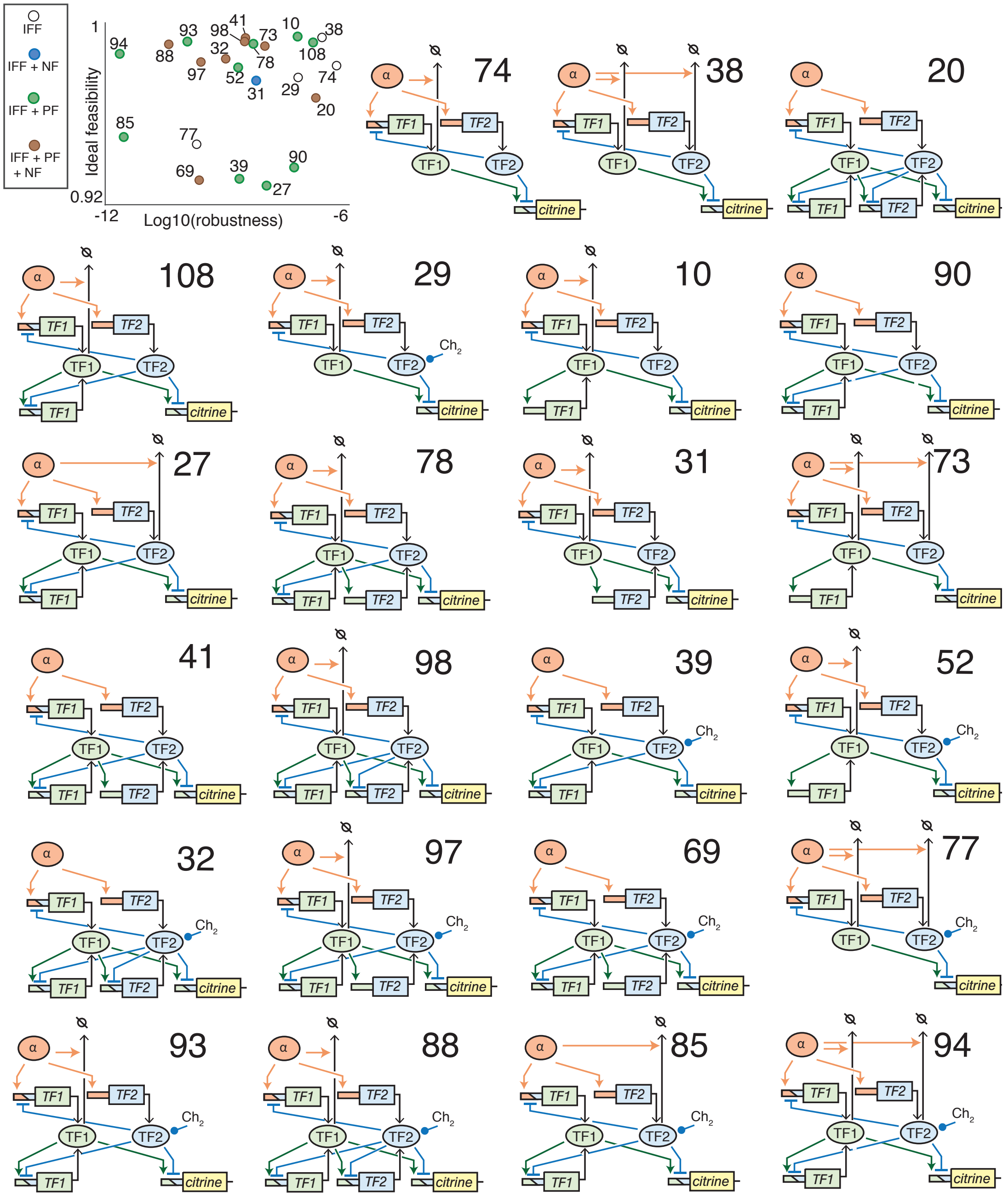

Supplementary Fig. 2 – Topologies with ideal feasibility > 0.9. Topologies are ordered by decreasing robustness (left to right). Symbols correspond to Fig. 1 of the main text.

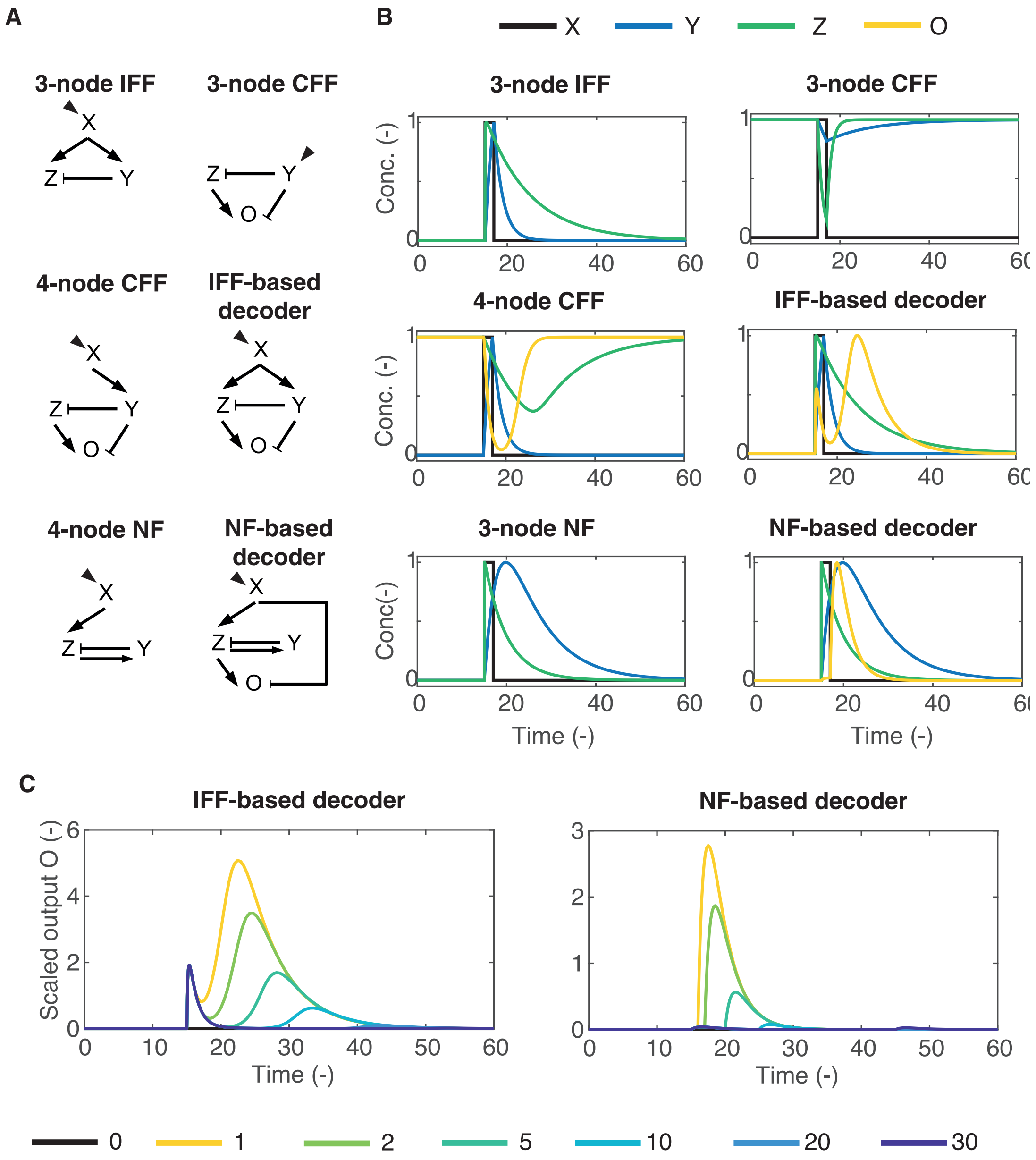

Supplementary Fig. 3 – Network motifs and their combination to functional decoders. **(A)** Network motifs and their composites comprising incoherent feedforward (IFF), coherent feedforward (CFF), and negative feedback (NF). The input node is indicated with a red triangle and it follows pulse dynamics. Note that the overall structure of the NF-based decoder is an IFF with embedded NF. **(B)** Simulated responses of simplified circuit models (see Supplementary Methods) for an input pulse of 2 A.U. duration. Adaptation to the input can be achieved by IFF or by (time-delayed) NF. CFF can produce a sign-sensitive delay in the response when the input is switched off. Combinations of motifs lead to output responses for the short pulse. Simulated concentrations are normalized by the trajectories' maxima for each state. **(C)** Simulation results for decoder outputs (node O in (A)) for varying pulse durations, demonstrating the ability to suppress output for long input pulses and without input. Colors indicate pulse durations (A.U. as in (B)).

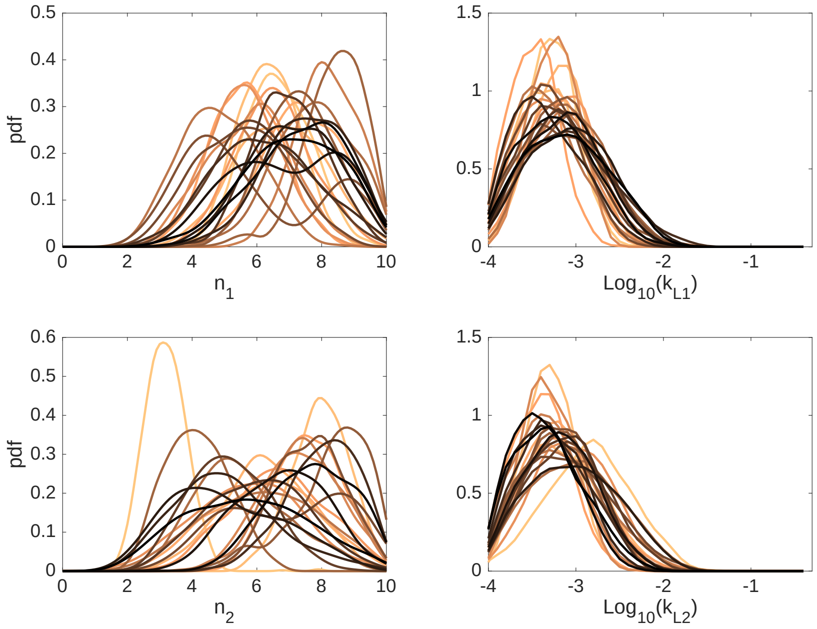

Supplementary Fig. 4 – Marginal distributions over the viable space for selected parameters for all topologies with ideal feasibility $>$ 0.9. 1,000 viable points were obtained by uniform sampling inside the viable region. Parameters: **(A)** Hill coefficient for TF1, **(B)** basal production rate with minimum induction by TF1, **(C)** Hill coefficient for TF2, **(D)** basal production rate with maximum repression by TF2. Exact parameter meanings and units are given in Supplementary Table 7. Lines are colored by robustness: darker is more robust.

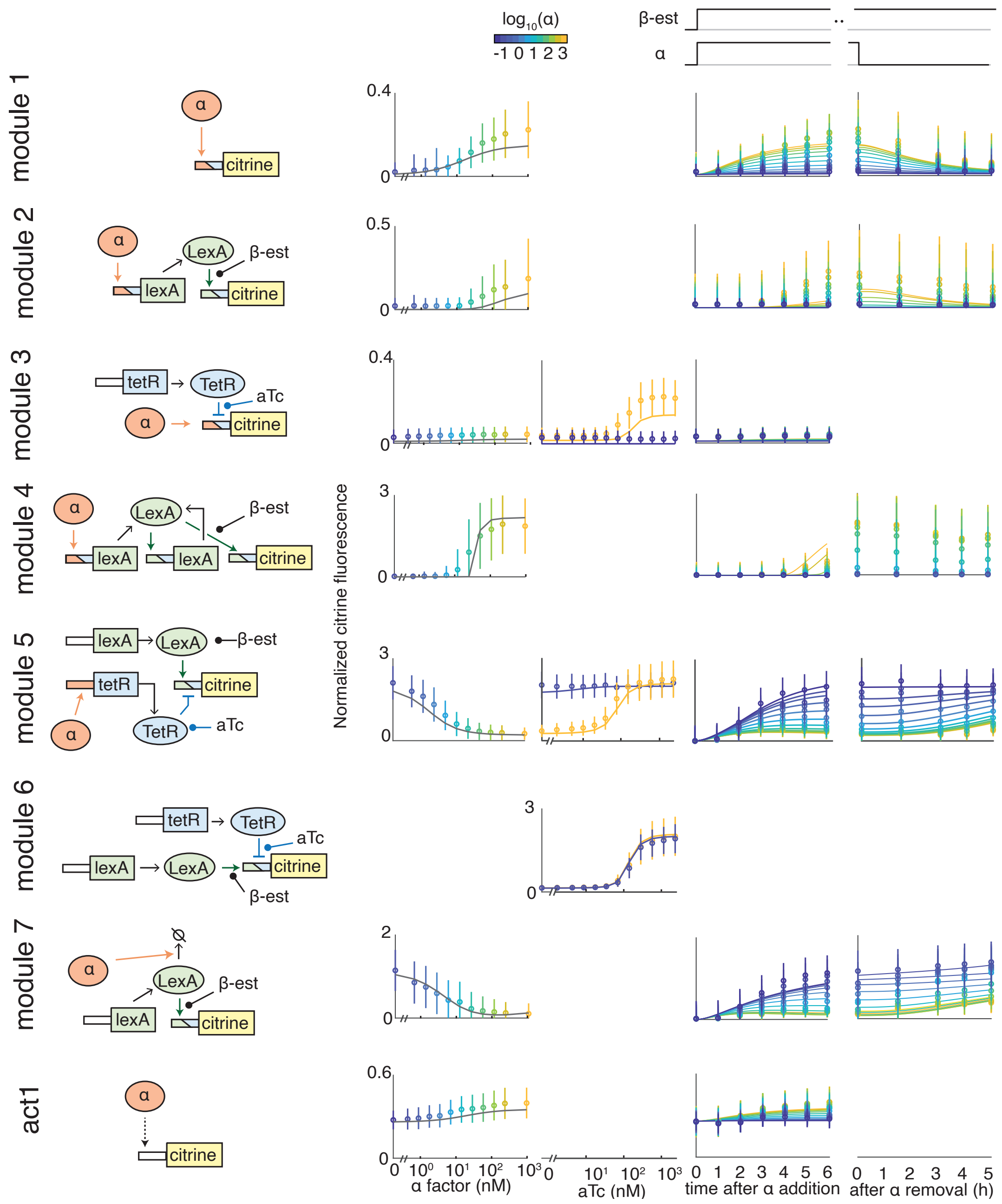

Supplementary Fig. 5 – Small informative synthetic networks (modules) measured to infer the parameter posterior. Strains for all modules are listed in Supplementary Table 2. All data points show normalized fluorescence measured by flow cytometry and include at least 4000 cells after gating. Symbols show experimental means,± standard deviation and lines simulations of the maximum likelihood parameter set estimated with all modules (χ^2 value is 96 for all modules when simulated without noise, and remains close to the χ^2 threshold of 1037 when noise is added to simulate each data point). Module 5 is also shown in Fig. 3. Each module was measured with relevant calibration experiments as described in Fig. 3 and in Methods. aTc dose responses were measured 6h after induction. When an α-factor release experiment was performed, the α-factor dose responses are shown at 18h, otherwise at 6h.

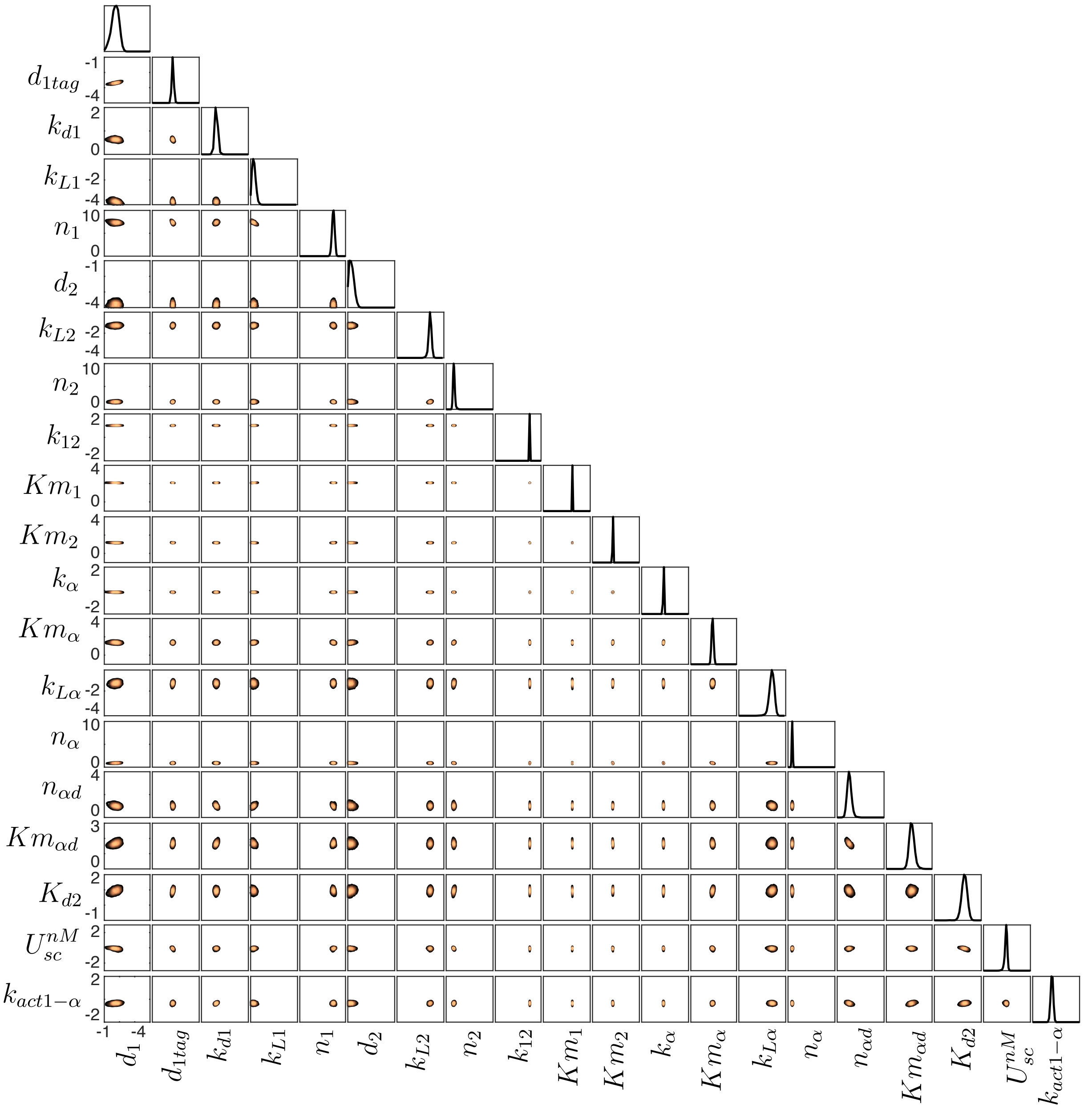

Supplementary Fig. 6 – Projection of the joint posterior parameter distribution on all possible pairs of parameters. Bright contour lines indicate high probability density. Parameter meanings, bounds and sampling mode (logarithmic or linear) are given in Supplementary Table 7 with a few adjustments detailed in the corresponding Supplementary Methods section.

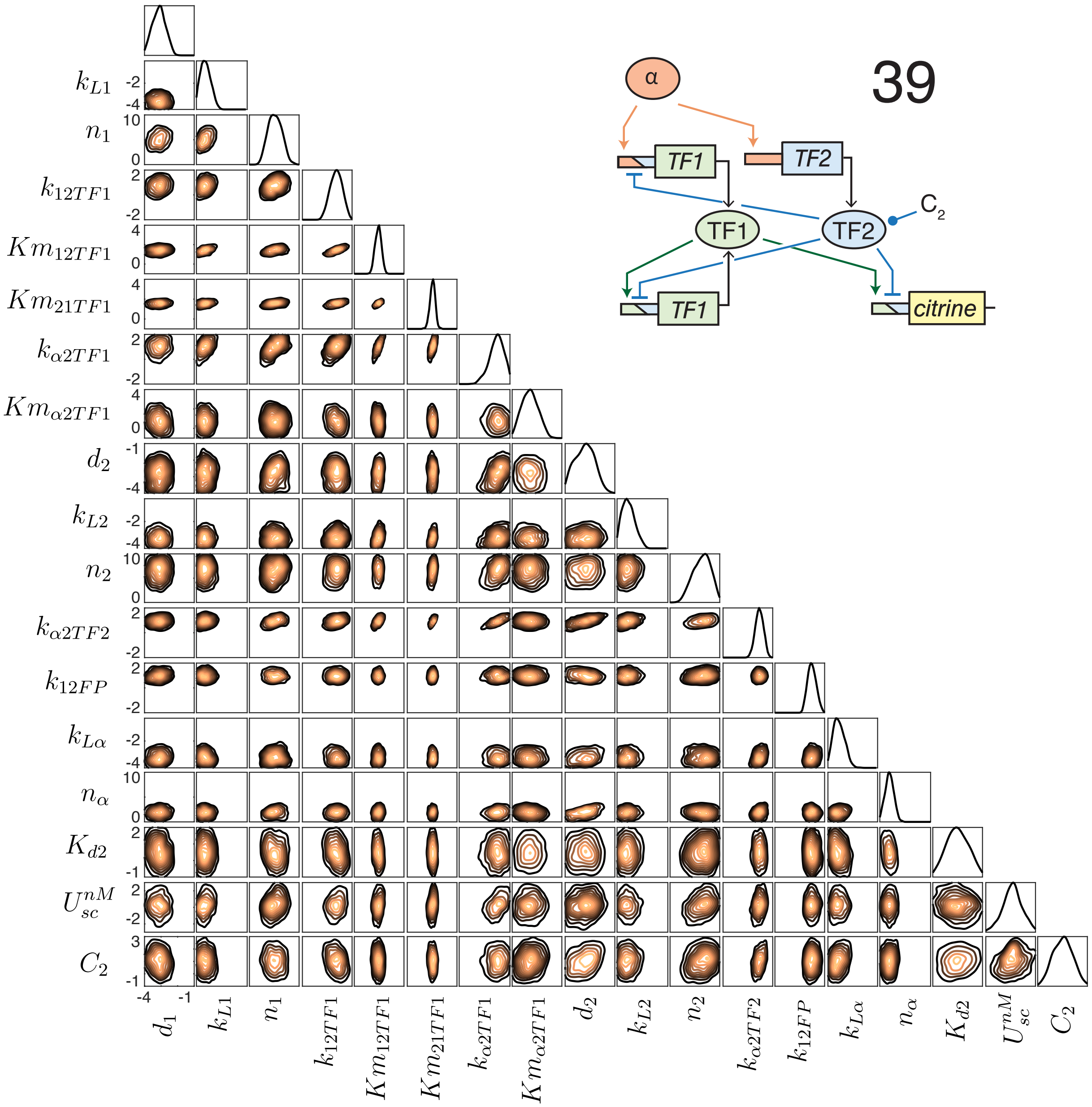

Supplementary Fig. 7 – Projection of the samples characterizing the viable space of topology 39 on all possible pairs of parameters. Bright contour lines indicate high density of projected samples (uniformly distributed in the multidimensional space). Parameter meanings, bounds and sampling mode (logarithmic or linear) are given in Supplementary Table 7.

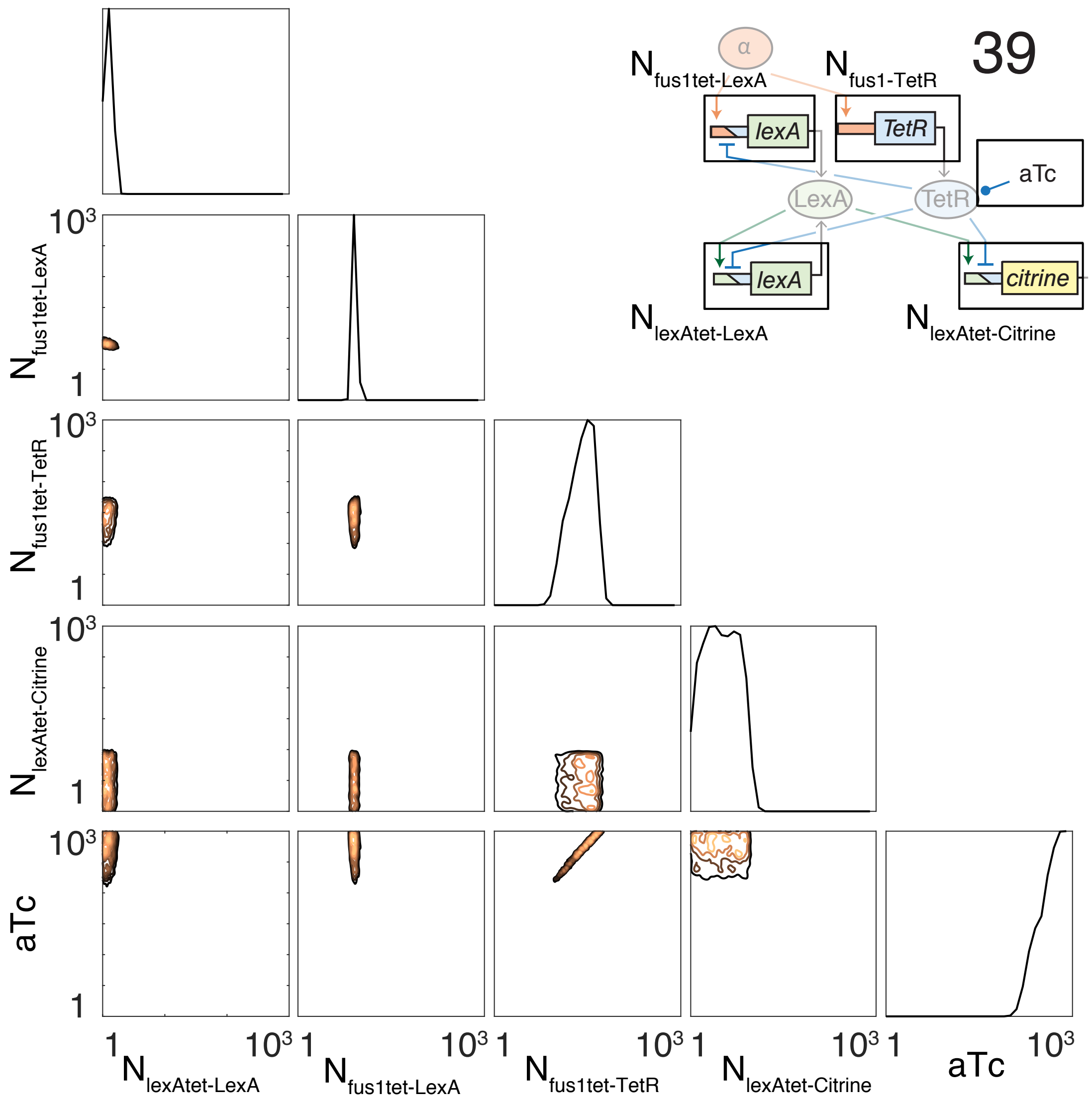

Supplementary Fig. 8 – Projection of the samples characterizing the high feasibility region on all possible pairs of tunable parameters for topology 39. Bright contour lines indicate high density of projected samples (uniformly distributed in the multidimensional space).

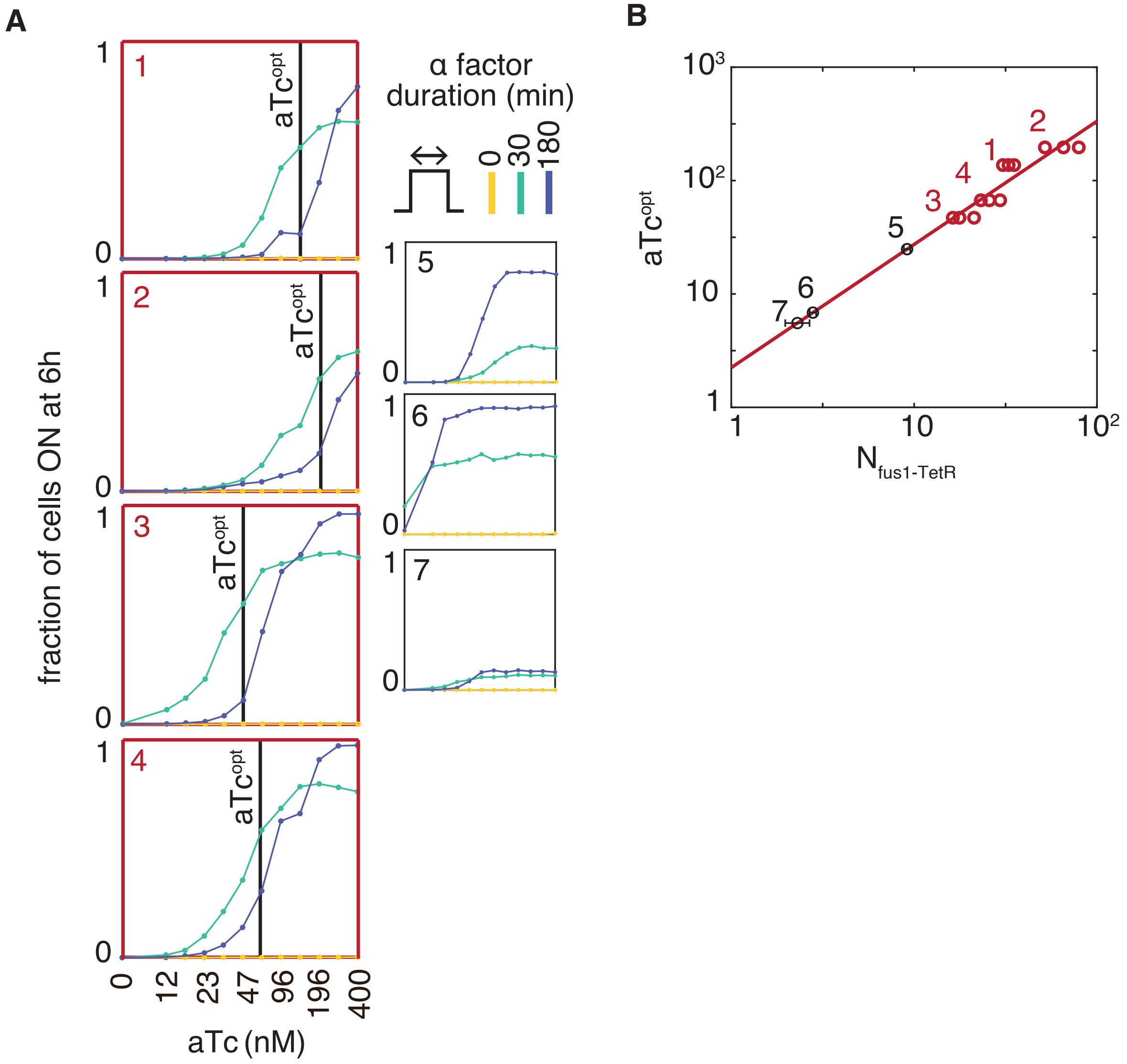

Supplementary Fig. 9 – Validation of the predicted decoder, results complementing Fig. 6. **(A)**. Responses at 6h to 0, 30 or 180 min pulses of 1μM alpha-factor for all circuit variants at varying aTc concentrations, used to determine an optimal aTc value (highest 30/180 min response ratio, with at least 50% of cells responding). All data points are measured by flow cytometry and include at least 4000 cells after gating. **(B)** Estimated values of optimal aTc for functional circuit variants (red) and extrapolated values for non functional variants (black). The extrapolation was based on the linear regression performed on all functional variants ($T_{39.1-4}$).

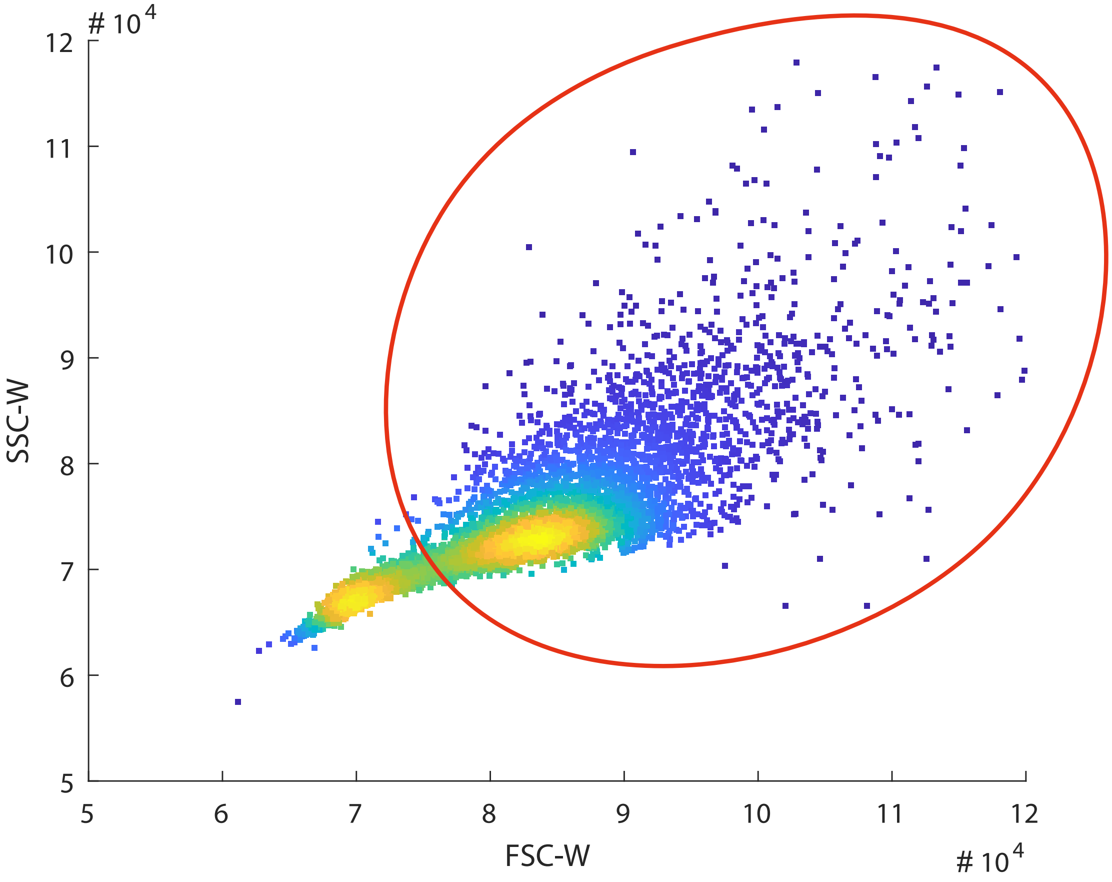

Supplementary Fig. 10 – Gating strategy.. In this work, we gated broadly budded cells using the signal width (W) parameter of forward (FSC) and side scatter (SSC). The exact same gate is always used for flow cytometry measurements appearing on the same plot.

Supplementary Methods

Topological Design Framework

Principle. TopoDesign relies on topological filtering[9;6;10], an efficient method to explore mechanistic model topologies and parameters at the same time, in a tractable manner, when enumeration would be too computationally intensive. The goal is to find which model topologies are able to either explain the data the best (for systems biology applications), or achieve a design objective the best (for synthetic biology applications as ours). Topologies that can achieve an objective are called viable. The starting topology is a complex topology from which all other topologies can be derived, by removing interactions. TopoFilter removes interactions by projecting parameters to particular values. For example, projecting the V_{max} to zero in a Michaelis-Menten rate law $V_{max}\frac{[S]}{[S]+Km}$ is equivalent to removing the action of the enzyme on that substrate. Briefly, TopoFilter starts by exploring the viable parameter space of this most complex topology with the efficient Hyperspace method[13], then checks for each viable parameter sample if it is still viable after projection of one of the parameters. If the answer is yes, we just found a viable point for a new topology. With this principle, TopoFilter focuses directly on the viable topologies and obtains viable samples for each of them without having to run a separate optimization each time.

Method inputs and definitions. TopoDesign requires a model for the starting topology, parameter specifications, and a cost function that defines the design objective.

Here, we consider parametric ordinary differential equation (ODE) models of the form:

$$\frac{dx(t)}{dt} = f(x(t), u(t), \theta), \quad x(t=0) = x_0,$$

where $x(t)$ is the vector of time-dependent system states (concentrations of molecular species), θ the vector of n_p model parameters (such as association and dissociation rate constants),

$u(t)$ the vector of (potentially) time-dependent inputs (such as ligand concentrations), and $f(x(t), u(t), \theta)$ the system of functions defining the rates of changes of the states. The initial conditions are given by x_0. Note that other types of parametric models, such as those describing stochastic dynamics, could be handled in the same framework.

Model parameters θ are constrained to the bounded, n_p-dimensional parameter space of the most complex model $\Theta \subset I\!R^{n_p}_{\geq 0}$, defined as $\Theta = [\theta_1^{min}, \theta_1^{max}] \times \cdots \times [\theta_{n_p}^{min}, \theta_{n_p}^{max}]$, where the upper and lower bounds for each parameter (indexed by subscripts) are problem-specific. One can assign them based on physical considerations (e.g., maximal rates of diffusion-limited reactions) or prior knowledge (e.g., on binding affinities).

For each parameter θ_k, we need to define if it can be projected, and if so, what its projection value is. At the projection value, a corresponding function in the model controlled by the parameter is inactivated and thereby generates a less complex topology. Note that dependencies between parameter projections may exist and need to be specified as well (see the decoder example in the next section). Correspondingly, a topology is defined by the subset of parameters that is not projected. Let the index set of all parameters in the most complex model be $\mathcal{I} = \{1, ..., n_p\}$. We denote the i-th topology as T_i and features of this topology by the superscript (i). For example, topology T_i is specified by the index set $\mathcal{I}^{(i)} \subseteq \mathcal{I}$; its total parameter space is $\Theta^{(i)} \subseteq \Theta$ and ${n_p}^{(i)}$-dimensional, with ${n_p}^{(i)} = |\mathcal{I}^{(i)}|$.

In addition, each parameter is designated as non-tunable, partially tunable, or fully tunable. Tunability is an intrinsic and quantitative property of a parameter, essentially reflecting the experimental effort associated with changing a parameter's value (in a given range). This property is hard to quantify in general (e.g., depending on parts availability if, for example, promoters are concerned where libraries of parts with different expression strength may exist, and depending on a particular lab's experimental capabilities).

Our classification determines how TopoDesign handles the different parameter classes:

(i) Fully tunable parameters: Experimentally, we have total control over these parameters, without molecular engineering (e.g., the aTc concentration). In TopoDesign, fully tunable parameters have no uncertainty. We can also vary them experimentally (to measure dose responses) during the small network characterization to obtain more information on the other parts. To compute the updated feasibility, which represents a best-case scenario after parameter tuning, we optimize fully tunable parameters.

(ii) Partially tunable parameters: Those parameters can be tuned quite easily by molecular engineering (e.g., copy numbers). Partially tunable parameters are associated with uncertainty; hence, they need to be in the set of estimated parameters. In addition, their values are optimized to compute the updated feasibility.

(iii) Non-tunable parameters: These are assumed to be fixed because of high experimental efforts for changing their values (e.g., affinities), their values need to be estimated, and they cannot be varied during the optimization to compute the updated feasibility.

We denote the index set of (partially or fully) tunable parameters by $\mathcal{T} \subseteq \mathcal{I}$.

Finally, cost functions capture if a model fulfills the design objectives or is consistent with the experimental data. Formally, a cost function $\mathcal{C}(\theta)$ evaluates if a particular topology achieves the target dynamic behavior for a parameter set θ. With a threshold ϵ, we consider parameter sets θ as viable if $\mathcal{C}(\theta) < \epsilon$. A topology is viable if we can obtain at least one viable parameter set for that topology. The viable space of T_i is then defined as $V_i = \{\theta^{(i)} \mid \mathcal{C}(\theta^{(i)}) < \epsilon\}$; it is approximated by sampling (see below for computational details). For concise notation, we also define a corresponding indicator function for viability,

$$\mathbf{1}_V(\theta^{(i)}) := \begin{cases} 1, & \text{if } \mathcal{C}(\theta^{(i)}) < \epsilon \\ 0, & \text{else.} \end{cases} . \tag{1}$$

Metrics. The robustness of a topology T_i is defined as in[6], that is, as the fraction of the total parameter space that is viable:

$$\mathcal{R}(T_i) = \frac{\mathrm{Vol}_{V_i}}{\mathrm{Vol}_{\Theta^{(i)}}}, \tag{2}$$

where Vol_{V_i} is the volume of the viable space,

$$\mathrm{Vol}_{V_i} = \int_{\Theta^{(i)}} \mathbf{1}_V(\theta^{(i)})\, d\theta^{(i)}$$

and $\mathrm{Vol}_{\Theta^{(i)}}$ is the volume of the total parameter space calculated as:

$$\mathrm{Vol}_{\Theta^{(i)}} = \prod_{k \in \mathcal{I}^{(i)}} (\theta_k^{max} - \theta_k^{min}).$$

The feasibility metric is defined differently from[6] because our previous metric did not capture the multidimensional shape of the viable region. The idea of the feasibility metric is to determine to what extent a probability distribution over a topology's parameters, $\pi(\cdot)$, is consistent with the viable space of the corresponding circuit when we can tune some or all of the parameters. We define feasibility as:

$$\mathcal{F}(T_i, \pi, \tau^{(i)}) = \int_{\Theta^{(i)}} \pi(\theta^{(i)} + \tau^{(i)}) \mathbf{1}_V(\theta^{(i)} + \tau^{(i)})\, d\theta^{(i)}. \tag{3}$$

Here, we represent shifted probability distributions in parameter space by a tuning vector $\tau^{(i)}$. Its components are: $\tau_k^{(i)} \in [\tau_k^{min}, \tau_k^{max}]$ if $k \in \mathcal{T}^{(i)}$ and $\tau_k^{(i)} = 0$ else. The possible tuning ranges $[\tau_k^{min}, \tau_k^{max}]$ are problem- but not topology-specific. Note that in the main text and figures, we simplified notation to integration over the viable space and represented (possibly tuned) probability distributions by $p_{\mathrm{x}}^{(i)}(\theta)$.

To represent the best-case scenario for a topology to be feasible, we compute ideal feasibility. Specifically, we check if we would be able to fit a parametrization of the model with minimal uncertainty in the viable space of the circuit, without additional constraints on parameter values (e.g., parameters of already defined parts). We approximate an ideally inferred

parameter posterior $\pi_{\text{ideal}}(\theta^{(i)})$ by a multivariate normal distribution with diagonal covariance matrix and a variance σ^2 and we optimize the location of the mean to compute

$$\mathcal{F}_{\text{ideal}}(T_i) = \max_{\tau^{(i)}} \mathcal{F}(T_i, \pi_{\text{ideal}}, \tau^{(i)}). \tag{4}$$

For optimization, the center of the distribution is initially positioned in the center of the viable space by computing the mean of viable samples. The uncertainty represented by σ can be estimated from data (see decoder application for a specific example). Then, we allow for tuning of all parameters over the entire space, that is, $\mathcal{T}^{(i)} = \mathcal{I}^{(i)}$.

Bayesian updates. With experimental data $\mathcal{D}$, we compute updated feasibilities

$$\mathcal{F}_{\text{updated}}(T_i) = \max_{\tau^{(i)}} \mathcal{F}(T_i, \pi_{\text{updated}}, \tau^{(i)}) \tag{5}$$

by using the estimated Bayesian parameter posterior, with $\pi_{\text{updated}}(\theta^{(i)}) = p(\theta^{(i)}|\mathcal{D})$ (see below for the approximation of the posterior). Note that the robustness metric $\mathcal{R}$ does not require updating because it is defined by the design objective and topology alone.

Computation. Computations rely on the approximate Bayesian computation (ABC) framework, in which the relevant quantities are determined by sampling in parameter space [11]. With an initial viable point for the most complex topology, the Matlab package TopoFilter generates sets of viable parameters that approximate each functional topology's viable space [9;6;10]. However, these samples are not uniformly distributed in the viable space, as required for the robustness metric. The samples are the output of the two first steps of the Hyperspace method [13]: an out-of-equilibrium adaptive Monte Carlo (OEAMC) sampling followed by a multiple ellipsoid-based sampling (MEBS). With these samples, the volume integration (`Volint`) function (third step of Hyperspace) computes simultaneously a large set of uniformly distributed viable points, an estimate of the viable volume, and this estimate's uncertainty. When the number of samples

from TopoFilter for a topology is too low for accurate integration, one can re-run the OEAMC and MEBS steps for that topology to generate sufficiently many samples.

Computations for determining the parameter posterior given a data set $\mathcal{D}$ follow the same principle of approximate Bayesian computation. Here, we adapted the simplest ABC rejection sampler algorithm from[11]. It consists of drawing uniform samples of the prior distribution, computing a distance function, and accepting only samples for which the distance is below a threshold. If the threshold is small enough, the output set of parameters is a good approximation of the posterior distribution $p(\theta|\mathcal{D})$.

Our distance function computes the sum of squares of the weighted residuals between simulated ($\hat{\mathcal{D}}_k(\theta)$) and experimentally measured ($\mathcal{D}_k$) data over all $n_d = |\mathcal{D}|$ data points k (this is another cost function, different from the one used in the design objective):

$$\mathcal{C}_\mathcal{D}(\theta) = \sum_{k=1}^{n_d} \left(\frac{\hat{\mathcal{D}}_k(\theta) - \mathcal{D}_k}{\sigma_k} \right)^2 ,$$

where σ_k is the experimentally observed standard deviation of the measurement for data point k. This requires a problem-specific measurement model to relate the model simulations for a given parameter set θ to the experimental observations (see decoder application for an example). Our threshold $\epsilon_\mathcal{D}$ is given by the χ^2 inverse cdf with $n_d - \hat{n_p}$ degrees of freedom, where $\hat{n_p} \leq n_p$ is the number of estimated parameters.

In high-dimensional parameter spaces, uniform sampling usually provides too few samples because there is a very low chance for a sample to fall in the high posterior probability region. We circumvent this problem by focusing only on high-probability regions. Only for the purpose of Bayesian parameter estimation, in order to keep the terminology of[13], we define as viable all parameter sets for which $\mathcal{C}_\mathcal{D}(\theta) < \epsilon_\mathcal{D}$, and assume that the probability density is equal to zero outside of the viable regions, allowing us to use the efficient parameter space characterization method from[13].

Synthetic decoder design

Model for the starting topology. Our ordinary differential equation (ODE) model involves an input u_α for the alpha-factor added in the medium and four states for the total concentrations of the activator TF_1, the repressor TF_2, the immature fluorescent protein FP_{imm}, and the mature fluorescent protein FP:

$$
\begin{aligned}
\frac{dTF_1}{dt} &= A_{P12-TF_1} + A_{P\alpha 2-TF_1} - D_{\alpha-TF_1} \cdot TF_1 \\
\frac{dTF_2}{dt} &= A_{P12-TF_2} + A_{P\alpha 2-TF_2} - D_{\alpha-TF_2} \cdot TF_2 \\
\frac{dFP_{imm}}{dt} &= A_{P12-FP_{imm}} + A_{P\alpha 2-FP_{imm}} - (k_{mat} + \rho) \cdot FP_{imm} \\
\frac{dFP}{dt} &= k_{mat} \cdot FP_{imm} - \rho \cdot FP.
\end{aligned}
$$

Here, $A_{Px2-ORF}$ is the production rate functions for the hybrid $P_{x/2}$ promoters inducible by x and repressible by TF_2, where $x \in \{\alpha, 1\}$ denotes the input (alpha-factor or TF_1) and $ORF \in \{TF1, TF2, FP_{imm}\}$ denote which protein's expression is controlled by the promoter. The function for turnover inducible by alpha-factor is $D_{\alpha-TFy}$, with $y \in \{1, 2\}$, the maturation rate constant of the FP is k_{mat} and its degradation (dilution) constant ρ.

The production rates are given by:

$$
A_{Px2-ORF} = k_{Px2-ORF} \left[k_{Lx} + (1 - k_{Lx}) \left(\mathcal{H}^{act}(X_{on}) \Big(k_{L2} + (1 - k_{L2}) \mathcal{H}^{rep}(TF_{2on}) \Big) \right) \right].
$$

They involve a maximal expression constant ($k_{Px2-ORF}$), constitutive terms for the basal expression (via k_{Lx} and k_{L2} for gene expression that cannot be repressed by TF_2), and controlled terms modeled by Hill functions $\mathcal{H}^{act}(X_{on})$ and $\mathcal{H}^{rep}(TF_{2on})$ that depend on the active concentrations of activators $X_{on} = \alpha$ or TF_{1on}, and on the active concentration of repressor TF_{2on}.

Activating Hill functions are defined as:

$$
\mathcal{H}^{act}(X_{on}) = \frac{X_{on}^{n_x} + \epsilon}{X_{on}^{n_x} + Km_{Px2-ORF}^{n_x} + \epsilon}.
$$

Projecting $Km_{Px2-ORF}$ to zero makes the inducible factor become constant, equal to one, and the small number ϵ ensures that this projection does not lead to numerical errors if the value of X_{on} is too small. The inducible part of the production rates can be repressed by TF_2, and the corresponding repressing Hill function is:

$$\mathcal{H}^{rep}(TF_{2on}) = \frac{Km_{P2x-TFy}^{n_2}}{TF_{2on}^{n_2} + Km_{P2x-TFy}^{n_2}}.$$

Projecting $Km_{P2x-TFy}$ to 10^8 makes the repressible factor become constant, equal to one, so that the promoter becomes only an inducible promoter.

We use a different $Km_{P2x-TFy}$ for each possible interaction instead of a single Km per transcription factor in order to be able to project all interactions independently in the topological filtering step, and generate all possible circuits.

The turnover rate functions for $y = 1$ or 2 are given by

$$D_{\alpha-TFy} = \left(d_y \frac{k_{dy}\alpha^{n_{\alpha d}} + Km_{\alpha d}^{n_{\alpha d}}}{\alpha^{n_{\alpha d}} + Km_{\alpha d}^{n_{\alpha d}}} + \rho \right),$$

where d_y is y's degradation rate constant in the absence of a degradation tag and ρ the dilution rate constant due to growth. d_y can be increased by a factor of k_{dy} if we add a degradation tag inducible by alpha factor (phospho-degron designed by[5]), with alpha-factor concentration α. This interaction can be removed by projecting the $Km_{\alpha d}$ value to 10^8. For FP and FP_{imm}, the degradation is negligible compared to ρ.

Fixed parameters are listed in Supplementary Table 6 and optimized parameters are given in Supplementary Table 7. Some projectable parameters directly lead to the removal of an interaction when they are projected, for example $k_{P12-TF1} \longrightarrow 0$ or $Km_{2\alpha TF2} \longrightarrow 10^8$. Some other parameters have an arbitrary projection value, such as $d_2 \longrightarrow 1$. These parameters are only projected as a consequence of other projections when they do not have any impact on the system anymore (therefore their projection value does not matter). In this case, d_2 would be projected only if all production rates producing TF_2 are projected. These implied projections

enable to prevent unnecessary sampling of parameters. All couplings and logic implications between parameter projections are listed in Supplementary Table 8.

All concentrations are expressed in act1 production units (apu), defined as the concentration produced by the act1 constitutive promoter in one minute in the absence of alpha factor. This unit enables to directly map our simulations to fluorescence data with a control measurement of the act1 constitutive strain (yCL106) in every data set. The variable TF_{1on} (resp. TF_{2on}) is the active concentration of TF_1 (resp. TF_2) after addition of an activating (resp. deactivating) chemical Ch_1 (resp. Ch_2). These chemicals are equivalent to β-estradiol and aTc in our final circuit.

We obtain the active concentrations TF_{1on} and TF_{2on} by assuming rapid equilibrium of the binding reactions below:

$$TF_{1free} + Ch_{1free} \longleftrightarrow TF_{1on} \Rightarrow K_{d1} = \frac{TF_{1free}Ch_{1free}}{TF_{1on}}$$
$$TF_{2on} + Ch_{2free} \longleftrightarrow TF_{2bound} \Rightarrow K_{d2} = \frac{TF_{2on}Ch_{2free}}{TF_{2bound}}.$$

In our starting topology, Ch_1 is present to allow for the use of regulated activators such as the LexA-ER-AD series from[8]. The chemical's concentration will always be constant and in excess so we can assume that the free chemical has the concentration of the chemical added in the medium: $Ch_1 = Ch_{1free}$. Mass conservation gives $TF_1 = TF_{1free} + TF_{1on}$, leading to:

$$TF_{1on} = \frac{Ch_1}{Ch_1 + K_{d1}} TF_1.$$

Contrary to Ch_1, Ch_2 is here a tuning chemical that we want to keep as a potential way to tune our system, in case it leads to a robust solution (as we finally did with aTc). Its concentration will not necessarily be high compared to TF_2. Mass conservation gives $TF_2 = TF_{2on} + TF_{2bound}$ and $Ch_2 = Ch_{2free} + TF_{2bound}$. We thus need to solve the following equa-

tion:

$$K_{d2} = \frac{TF_{2on}(Ch_2 - (TF_2 - TF_{2on}))}{TF_2 - TF_{2on}}$$

to obtain:

$$TF_{2on} = \frac{1}{2}\Big(TF_2 - K_{d2} - Ch_2 + \sqrt{TF_2^2 + K_{d2}^2 + Ch_2^2 + 2K_{d2}(TF_2 + Ch_2) - 2Ch_2TF_2}\Big).$$

In order to have a correspondence to the experimental setup, we express Ch_1, Ch_2, K_{d1} and K_{d2} in nM rather than in act1 production units. We introduce the unit scaling parameter U_{sc}^{nM} so that we have for each variable : $X[nM] = X[apu] \times U_{sc}^{nM}$.

Cost function for the short pulse decoder objective. Our cost function is a mathematical formulation of the short pulse decoder design objective. It involves the response $FP(\theta, t, u_{\alpha,\Delta_t})$ to a pulse of 1μM alpha-factor of $\Delta_t = 0, 30$ or 180 minutes duration starting at $t = 0$ min within a 6 hours experiment ($t \in [0, 360]$). The alpha-factor pulse starts after a 400 min period to reach an initial steady-state. The cost function is given by

$$\mathcal{C}(\theta) = \max(\mathcal{C}_{basal}(\theta)^2, \mathcal{C}_{detect}(\theta)^2, \mathcal{C}_{30vs0}(\theta)^2, \mathcal{C}_{180vs30}(\theta)^2),$$

where each component accounts for one design constraint:

1. To ensure a basal level that does not exceed the order of magnitude of the steady-state FP concentration produced by the constitutive promoter act1, FP_{act1}^{SS}, when there is no input,

$$\mathcal{C}_{basal}(\theta) = \frac{FP(\theta, 360, u_{\alpha,0})}{100} < 1.$$

   The denominator (in act1 production units) is the order of magnitude derived by setting the following ODEs to zero:

$$\frac{dFP_{imm}}{dt} = k_{act1} - (k_{mat} + \rho)FP_{imm}$$
$$\frac{dFP}{dt} = k_{mat}FP_{imm} - \rho FP.$$

With k_{mat} and ρ values from Supplementary Table 6 and $k_{act1} = 1$ by definition, we obtain $FP^{SS}_{act1} = \frac{k_{mat}}{\rho + k_{mat}} \frac{k_{act1}}{\rho} \sim 90$ apu.

2. To ensure a response with a detectable output when there is a short input, we constrain the cost with the 20 and 30 minutes short inputs in order to prevent optimizing the cost finely for one particular duration:

$$\mathcal{C}_{detect}(\theta) = \max\left(\frac{100}{FP(\theta, 360, u_{\alpha,20})}, \frac{100}{FP(\theta, 360, u_{\alpha,30})}\right) < 1.$$

3. To ensure at least a 20-fold response for 30 min input vs no input,

$$\mathcal{C}_{30vs0}(\theta) = 20\frac{\max\limits_{t\in[0,360]}(FP(\theta, t, u_{\alpha,0}))}{FP(\theta, 360, u_{\alpha,30})} < 1.$$

4. Similarly, to ensure at least a 20-fold response for 30 min input vs 3h input,

$$\mathcal{C}_{180vs30}(\theta) = 20\frac{\max\limits_{t\in[0,360]}(FP(\theta, t, u_{\alpha,30}))}{FP(\theta, 360, u_{\alpha,180})} < 1.$$

All constraints are achieved if and only if $\mathcal{C}(\theta) < 1$, that is, $\epsilon = 1$ is used in Eq. 1 to classify a parameter set θ as viable.

Topological filtering We ran TopoFilter starting from a set of 100 initial viable points. We found these initial points by using a lattice of 100 random points sampled with latin hypercube (Matlab `lhsdesign` function) and running a global optimization for each of these points separately (enhanced scatter search method from MEIGO by[3]). We used 50,000 parameter samples for the initial topology sampling and used the option to re-sample viable topologies in their own parameter subspace if fewer than 20,000 viable samples were obtained. We obtained a set of 225 viable topologies (in the form of a list of projected parameters for each topology), and a set of viable parameter samples for each topology.

In order to enable independent projections of the different $Km_{P2x-TFy}$ and $Km_{Px2-TFy}$, we used a different parameter for each of them. However, there is in practice only one value Km_2 for all $Km_{P2x-TFy}$ (resp. Km_1 for all the $Km_{P12-TFy}$ and Km_α for all the $Km_{P\alpha 2-TFy}$) that captures the binding affinity of the transcription factors to the promoters, independent of the gene transcribed. Therefore, we reduced the obtained parameter sets by keeping the ones that are still viable after setting, for each parameter set, the $Km_{P2x-TFy}$, $Km_{P12-TFy}$, $Km_{P\alpha 2-TFy}$ values equal to one of them, now denoted as Km_2, Km_1 and Km_α. For some topologies, none of the new parameter samples was viable. We ended up with 109 viable topologies after this step.

Computing robustness and ideal feasibility. To approximate the viable regions, we used TopoFilter to obtain sets of $\sim$1000-2000 non-uniformly sampled viable parameters. We ran the Hyperspace `Volint` function after reducing the viable parameter samples to the same Km subspace (as explained above). When the Km reduction step resulted in too few samples for a topology, we re-ran the OEAMC and MEBS steps for that topology in the reduced parameter space to provide a sufficient input for the `Volint` function. We ran `Volint` with 5000 samples, got an average volume estimation accuracy of 4%, and used the estimated volume to determine $\mathcal{R}$.

To compute ideal feasibility, we approximated an ideally inferred parameter posterior by 5000 samples drawn from a multivariate normal distribution (in the log parameter space of the topology T_i) $p_{\text{ideal}}(\theta^{(i)})$ with diagonal covariance matrix, with a deviation $\sigma = log_{10}(2)/4$ and a center μ initially set as the median of the uniformly distributed viable samples. We used global optimization(enhanced scatter search method from MEIGO by[3]) to optimize the location of μ and compute $\mathcal{F}_{\text{ideal}}(T_i)$ in Eq. 4.

To estimate σ of an ideally inferred parameter distribution, we computed the posterior dis-

tribution for 7 parameters with the dose response data of a TetR-repressible promoter to aTc, PlexAtet (strain yCL110 described as module 6 in Supplementary Table 2, data shown in Supplementary Fig. **5**), in the presence of a saturating β-estradiol concentration (5 μM) and in the absence of alpha-factor. The modeling and parameter posterior computation were done exactly as described in the next section. The 7 inferred parameters are all parameters that concern the aTc repression of the PlexAtet promoter: $k_{12}, k_{L2}, Km_{21}, n_2, U_{sc}^{nM}, K_{d2}, d_2$. The width (4σ) of the log parameter marginal for the best inferred parameter k_{12} is on the order of $\log_{10}(2)$, meaning that even for a well inferred parameter, we can estimate its value only up to a factor 2.

Small informative networks for rapid prototyping. To estimate the parameters of our selected biological parts, we experimentally characterized the seven small informative networks (called modules) shown in Supplementary Fig. **5**, and used all the data in Supplementary Fig. **5** and in Fig. 3, except the alpha factor release dynamic data for module 4 (due to a high bimodality in the data that cannot be captured with our single-cell model). The models used to simulate the modules are mainly subsets of our general model for the starting topology and can thus be obtained by projecting parameters. LexA-ER-B112 replaces TF_1, TetR-NLS-MBP replaces TF_2, and Citrine replaces FP. Together, the seven modules involve all the parameters in Supplementary Table 7, except that a single Hill constant Km is allowed per transcription factor, and a single production rate k is allowed per promoter:

- a single Km_2 parameter is replacing all the $Km_{P2x-TFy}$
- a single Km_1 parameter is replacing all the $Km_{P1x-TFy}$
- a single Km_α parameter is replacing all the $Km_{P\alpha2-TFy}$
- a single k_{12} parameter is replacing all the $k_{P12-TFy}$
- a single $k_{\alpha2}$ parameter is replacing all the $k_{P\alpha2-TFy}$

- this time Ch_2 (i.e., aTc) is not optimized; it is instead a control parameter whose value is given by the experimental setup.

The setup for rapid prototyping involves two additional parameters. The constitutive expression of a construct by the act1 promoter in some modules leads to another production term in addition to $A_{Px2-ORF}$:

$$k_{act1} + k_{act1-\alpha}\left(k_{L\alpha} + (1-k_{L\alpha})\frac{\alpha^{n_\alpha}}{Km_\alpha^{n_\alpha} + \alpha^{n_\alpha}}\right).$$

By definition, $k_{act1} = 1$ apu. $k_{act1-\alpha}$ is added to account for a small increase in the act1 promoter expression linked to the presence of α-factor that we observed experimentally. The other additional parameter is a different value for d_1 called d_{1tag} when TF_1 is lexA-ER-B112-phosphodegron (instead of lexA-ER-B112). We observed that the tagged LexA protein seems to degrade at a different rate than the original one. Indeed, this is the most probable way to explain that the response of module 7 (involving the lexA-ER-B112-phosphodegron) in the absence of alpha-factor is lower than the response of modules 5 and 6 without alpha-factor and with maximum aTc (Supplementary Fig. **5**).

Computing the parameter posterior. Our measurement model relates simulated and measured fluorescence intensities for each experimental observation k to each other via:

$$\hat{\mathcal{D}}_k(\theta) = \mathcal{D}^{\text{empty}} + U_{sc}^{Fluo} FP_k(\theta) + e_k, \;\; e_k \sim \mathcal{N}(0, \sigma_k^2),$$

where $\hat{\mathcal{D}}_k(\theta)$ is the simulated normalized fluorescence for this data point, $\mathcal{D}^{\text{empty}}$ is the mean autofluorescence observed with an empty strain, e_k is the measurement noise, assumed to be normally distributed with the observed standard deviation σ_k, $FP_k(\theta)$ is a short-hand notation for fluorescent protein concentration at time t_k, and U_{sc}^{Fluo} is a unit scaling factor that converts act1 production units into normalized fluorescence units. This factor is calculated for each data set with the observed mean fluorescences of the act1 and empty strains in the absence of alpha

factor as follows:

$$U_{sc}^{Fluo} = \frac{\mathcal{D}^{\text{act1}} - \mathcal{D}^{\text{empty}}}{FP_{act1}^{SS}} \text{ with } FP_{act1}^{SS} = \frac{k_{mat}}{\rho(\rho + k_{mat})}.$$

For the derivation of FP_{act1}^{SS}, see the section on the cost function for the short pulse decoder objective.

The efficient parameter space characterization method from[13] enabled us to obtain more than 5,000 samples of the posterior distribution by sampling uniformly inside a good estimation of the viable region with respect to the data. Projections of the 20-dimensional posterior distribution are shown in Supplementary Fig. **6**. Note that we did not estimate fully tunable parameters such as the aTc concentration.

Updating the feasibility metric and computing the high-feasibility region. To update the feasibility metric, we first needed to map the parameter posterior $p(\theta|\mathcal{D})$ to the same parameter space for each topology T_i, to generate the mapped $p(\theta^{(i)}|\mathcal{D})$. We achieved this by removing from the posterior the parameters that are not involved in the corresponding topology (using $\mathcal{I}^{(i)}$), adding an arbitrary value of 1 for fully tunable parameters to all parameter sets, and duplicating the values of the k_{P12} and $k_{P\alpha 2-}$ as many times as the number of constructs in the topology required.

Note that for parameters optimized and sampled in log space, the tuning factors τ are also in log space, so tuning a parameter this way corresponds to multiplying the parameter value by the tuning factor.

For our short pulse decoder, we first computed the updated feasibility for all topologies without tuning any parameter, and obtained a zero feasibility because it is very unlikely that the parts measured directly fall into the correct region of the parameter space. We then decided to select the aTc concentration as a fully tunable parameter, and maximum production rates $k_{Px2-TFy}$ as partially tunable parameters. In this case, the non-zero elements of the tuning

parameter vector τ were the concentration of aTc and the number of copies of each promoter of the circuit, $N_{Px2-TFy}$. The logarithmic bounds used for optimization were $\tau_k^{\text{min}} = 0$ and $\tau_k^{\text{max}} = 3$ in all cases.

The high-feasibility region for circuit 39 (Fig. 6B) was obtained by computing the regions spanned by $\tau^{(39)}$ where $\mathcal{F}(T_{39}, \pi_{updated}, \tau^{(39)}) \geq 0.9\, \mathcal{F}_{updated}(T_{39})$ (with $\mathcal{F}$ and $\mathcal{F}_{updated}$ defined in 3 and 5) with the Hyperspace toolbox[13]. In order to plot the region with a line instead of points, we took the outer line generated by the `contourf` function in Matlab.

Population predictions for circuit T_{39} To simulate the population response to a pulse of alpha factor as shown in Fig. 6D, we sampled 1000 parameter vectors from the posterior distribution (see section "Computing the parameter posterior distribution") and simulated circuit T_{39}^{sim} with this parameter vector, and with its optimal copy numbers and aTc concentration displayed in Fig. 6B. We simulated the response to no input, to a 30min alpha-factor pulse and to a 180min pulse for each of the 1000 parameter vectors. The response is a predicted Citrine concentration FP. In order to predict the observed normalized log-fluorescence displayed together with the observed data, we used a similar measurement model as the one for computing the parameter posterior:

$$\log \mathcal{D}^{sim}(\theta) = \log(\mathcal{D}^{\text{empty}} + U_{sc}^{Fluo} FP(\theta)) + e, \;\; e \sim \mathcal{N}(0, \sigma^2),$$

We computed the scaling factor U_{sc}^{Fluo} as before, based on the measurements of the mean normalized fluorescence of the empty and act1 control strains measured together with the data observed in Fig. 6D. However, contrary to the model used for computing the parameter posterior, it was not possible to use a different σ_k based on the data for each observation since we did not measure these data - we were making predictions. We assumed instead a constant lognormal noise that we estimated using the deviation observed for the log-fluorescence of the empty strain $\sigma = sd(\log(\mathcal{D}^{\text{empty}}))$.

To simulate the population response for each simulated dot in Fig. 6A, we did as for Fig. 6D, but replaced the optimal aTc by the corresponding dose used in the experiment, and used the same threshold for ON cells as for processing the experimental data (threshold such that the unimodal response of control circuit C_3 to a 3h pulse has 95% cells ON).

Network motifs and their combination to functional decoders

To analyze design principles at the core of functional short pulse decoders, and to investigate alternatives to the architectures found by TopoDesign for our problem specification, we used simplified dynamic models for network motifs. Supplementary Fig. **3**A shows the network motifs and their combinations that we considered. Networks comprise a subset of three internal states (X,Y, and Z) and an output state (O) that is controlled by AND logic. An external input signal to a node is denoted by $S(t)$ and the corresponding node dynamics follows the input directly. Model definitions for motifs follow the work by Mangan and Alon on feedforward motifs[7].

Regulation functions and inputs. We use Hill kinetics with Hill coefficients H and apparent affinities K_{ij} for node i controlling node j to define regulation functions. For the activating function

$$f^{+}(u, K, H) = \frac{(u/K)^H}{1 + (u/K)^H},$$

where u denotes the effector node's state and K the affinity. Similarly, the repressing function is defined by:

$$f^{-}(u, K, H) = \frac{1}{1 + (u/K)^H}.$$

A pulse input with start time t_0 and duration τ is captured as:

$$S(t) = \begin{cases} 1 & \text{if } t_0 \le t \le t_0 + \tau \\ 0 & \text{otherwise} \end{cases}.$$

Incoherent feedforward (IFF). The ODEs for IFF-based circuits are defined as:

$$\begin{aligned}
X(t) &= S(t) \\
\frac{dY(t)}{dt} &= B_y + \beta_y \cdot f^{+}(X(t), K_{xy}, H) - d_y \cdot Y(t) \\
\frac{dZ(t)}{dt} &= B_z + \beta_z \cdot f^{+}(X(t), K_{xz}, H) \cdot f^{-}(Y(t), K_{yz}, H) - d_z \cdot Z(t) \\
\frac{dO(t)}{dt} &= B_o + \beta_o \cdot f^{+}(Z(t), K_{zo}, H) \cdot f^{-}(Y(t), K_{yo}, H) - d_o \cdot O(t),
\end{aligned}$$

where B_i denote basal expression rates, β_i maximal expression rate constants, and d_i degradation constants for nodes i. The first two equations define the IFF motif, the complete ODE system the IFF-based decoder.

Coherent feedforward (CFF). The ODE system for the three-node CFF is:

$$\begin{aligned}
Y(t) &= S(t) \\
\frac{dZ(t)}{dt} &= B_z + \beta_z \cdot f^{-}(Y(t), K_{yz}, H) - d_z \cdot Z(t) \\
\frac{dO(t)}{dt} &= B_o + \beta_o \cdot f^{+}(Z(t), K_{zo}, H) \cdot f^{-}(Y(t), K_{yo}, H) - d_o \cdot O(t),
\end{aligned}$$

and for the extended (four-node) CFF, similarly, we have:

$$\begin{aligned}
X(t) &= S(t) \\
\frac{dY(t)}{dt} &= B_y + \beta_y \cdot f^{+}(X(t), K_{xy}, H) - d_y \cdot Y(t) \\
\frac{dZ(t)}{dt} &= B_z + \beta_z \cdot f^{-}(Y(t), K_{yz}, H) - d_z \cdot Z(t) \\
\frac{dO(t)}{dt} &= B_o + \beta_o \cdot f^{+}(Z(t), K_{zo}, H) \cdot f^{-}(Y(t), K_{yo}, H) - d_o \cdot O(t).
\end{aligned}$$

Negative feedback (NF). Analogous to the IFF-based circuits, the first two ODEs define the NF motif, the entire system defines the NF-based decoder:

$$
\begin{aligned}
X(t) &= S(t) \\
\frac{dY(t)}{dt} &= B_y + \beta_y \cdot f^+(Z(t), K_{zy}, H) - d_y \cdot Y(t) \\
\frac{dZ(t)}{dt} &= B_z + \beta_z \cdot f^+(X(t), K_{xz}, H) \cdot f^-(Y(t), K_{yz}, H) - d_z \cdot Z(t) \\
\frac{dO(t)}{dt} &= B_o + \beta_o \cdot f^+(Z(t), K_{zo}, H) \cdot f^-(X(t), K_{xo}, H) - d_o \cdot O(t).
\end{aligned}
$$

Note that the model for the NF-based decoder is minimal and not necessarily biologically realistic without including additional components: regulator X has to act simultaneously as activator and repressor. TopoDesign, correspondingly, did not include this circuit architecture.

Model parameters. Default parameter values for all circuits were $B_y = B_z = B_o = 0$, $H = 2$, $\beta_y = \beta_z = \beta_o = 1$, $d_y = 0.5$, $d_z = 0.1$, $d_o = 1$, $K_{xy} = K_{xz} = K_{xo} = K_{zo} = K_{yo} = 0.1$, and $K_{yz} = 0.01$. For NF-based circuits, we used $K_{yz} = 0.001$, $K_{zy} = 0.01$, $d_y = 0.125$, and $d_z = 0.2$. Parameters were set manually to illustrate the qualitative dynamics; optimized values could, for example, reduce the initial output response upon stimulation (Supplementary Fig. 3BC) as in the models identified by TopoDesign.

Supplementary Tables

Supplementary Table 1 – Plasmids used in this work. Backbone IDs refer to[4].

ID	Backbone ID	Insert	Source
FRP1353	pRG207	Pact1-citrine-tCyc1	previous work[4]
FRP1474	pRG201	Pact1-lexA-ER-B112-tCyc1	previous work, based on FRP880 in[8]
FRP2306	pRG203MX	Pact1-tetR-nls-malE-tCyc1	[1]
pCL12	pRG207	PlexAtet-citrine-tCyc1	this work
pCL21	pRG201	Pact1-lexA-ER-B112-phosphodegron-tCyc1	this work. part of the insert from[5]
pCL27	pRG207	Pfus1tet-citrine-tCyc1	this work
pCL28	pRG206MX	PlexAtet-lexA-ER-B112-tCyc1	this work
pCL33	pRG203MX	Pfus1mut-tetR-nls-malE-tCyc1	this work
pCL38	pRG235	Pfus1tet-lexA-ER-B112-tCyc1	this work
pCL66	pRG235	Pfus1mut-tetR-nls-malE-tCyc1	this work

Supplementary Table 2 – Yeast strains used in this work.

ID	Name	Content	Genotype
FRY69 [4]		-	BY4743, MATa, met15Δ0 his3Δ1 leu2Δ0 ura3Δ0 lys2Δ0, FRP235::tAdh1
yCL102	empty	-	FRY69, bar1::Nat, far1::KanMX
yCL103	module 1	Pfus1tet-Citrine	yCL102, pCL27::Lys2
yCL104	module 2	Pfus1tet-lexA-ER-B112<br>PlexAtet-Citrine	yCL102, pCL26::Met15, pCL12::Lys2
yCL105	module 3	Pact1-tetR-nls-malE<br>Pfus1tet-Citrine	yCL102, FRP2306::His3, pCL27::Lys2
yCL106	act1	Pact1-Citrine	yCL102, FRP1353::Lys2
yCL107	module 4<br>or<br>control circuit C_2	Pfus1tet-lexA-ER-B112<br>PlexAtet-lexA-ER-B112<br>PlexAtet-Citrine	yCL102, pCL26::Met15, pCL28::Ura3, pCL12::Lys2
yCL109	module 5	Pact1-lexA-ER-B112<br>Pfus1mut-tetR-nls-malE<br>PlexAtet-Citrine	yCL102, FRP1474::Met15 pCL33::His3, pCL12::Lys2
yCL110	module 6	Pact1-lexA-ER-B112<br>Pact1-tetR-nls-malE<br>PlexAtet-Citrine	yCL102, FRP1474::Met15, FRP2306::His3, pCL12::Lys2
yCL113	module 7	Pact1-lexA-ER-B112-phosphodegron<br>PlexAtet-Citrine	yCL102, pCL21::Met15, pCL12::Lys2
yCL114	control circuit C_1	PlexAtet-lexA-ER-B112<br>PlexAtet-Citrine	yCL102, pCL28::Ura3, pCL12::Lys2
yCL125	control circuit C_3	Pfus1tet-lexA-ER-B112 (23 copies)<br>PlexAtet-lexA-ER-B112<br>PlexAtet-Citrine	yCL102, pCL38::Ty1δ, pCL28::Ura3, pCL12::Lys2
yCL130-133, 141-143	T39.1-7	Pfus1mut-tetR-nls-malE (multicopy)<br>Pfus1tet-lexA-ER-B112 (multicopy)<br>PlexAtet-lexA-ER-B112<br>PlexAtet-Citrine	yCL102, pCL66::Ty1δ, pCL38::Ty1δ, pCL28::Ura3, pCL12::Lys2

Supplementary Table 3 – Sequences of promoters assembled in a modular way for this work. We highlight the core promoter sequence of P2tet from[1] (brown), lexA operators (violet), tet operators (blue). For Pfus1mut, the tet operators (cyan) are shuffled to prevent binding of TetR on the Pfus1mut promoter, while keeping the same properties as Pfus1tet.

PlexAtet	tatatttcttttcgcggccgcctatagggcgaattggagctccctagg<br>tgctgtatatactcacagcataactgtatatacacccagggtctagg<br>tgctgtatatactcacagcataactgtatatacacccagggtctagg<br>tgctgtatatactcacagcataactgtatatacacccagggtctagg<br>tgctgtatatactcacagcataactgtatatacacccagggtctaga<br>aaaggttgaaaccagttccctgaaattattcccctatctatcattgatagatataaaagtctatcattgatagagtaattc<br>tgtaaatctatttcttaaacttcttaaattctacttttatagttagtctttttttttagttttaaaacaccaagaacttagt<br>ttcgaataaacacacataaacaaacaaa
Pfus1tet	acgatgattcagttcgccttctatcctttgtttacgtatttgtttatatatataactttatttttttttattaattgggctg<br>caagacaattttgttgtcagtgatgcctcaatccttcttttgcttccatatttaccatgtggaccctttcaaaacagagttg<br>tatctctgcaggatgcccttttttgacgtattgaatggcataattgcactgtcacttttcgcgctgtctcattttggtgcgat<br>gatgaaacaaacatgaaacgtctgtaatttgaaacaaataacgtaattctcgggattggttttatttaaatgacaatgtaag<br>agtggctttgtaaggtatgtgttgctctaga<br>aaaggttgaaaccagttccctgaaattattcccctatctatcattgatagatataaaagtctatcattgatagagtaattc<br>tgtaaatctatttcttaaacttcttaaattctacttttatagttagtctttttttttagttttaaaacaccaagaacttagt<br>ttcgaataaacacacataaacaaacaaa
Pfus1mut	acgatgattcagttcgccttctatcctttgtttacgtatttgtttatatatataactttatttttttttattaattgggctg<br>caagacaattttgttgtcagtgatgcctcaatccttcttttgcttccatatttaccatgtggaccctttcaaaacagagttg<br>tatctctgcaggatgcccttttttgacgtattgaatggcataattgcactgtcacttttcgcgctgtctcattttggtgcgat<br>gatgaaacaaacatgaaacgtctgtaatttgaaacaaataacgtaattctcgggattggttttatttaaatgacaatgtaag<br>agtggctttgtaaggtatgtgttgctctaga<br>aaaggttgaaaccagttccctgaaattattcccctactattcattgaatagtataaaagctattcattgaataggtaattc<br>tgtaaatctatttcttaaacttcttaaattctacttttatagttagtctttttttttagttttaaaacaccaagaacttagt<br>ttcgaataaacacacataaacaaacaaa

Supplementary Table 4 – Primers used in this work (except qPCR primers in supp. table 5).

ID	target	used for the assembly of	Direction	Sequence
FRO114	end of insert in pRG backbones [4]	modular cloning of all plasmids	reverse	AATTAACCCTCACTAAAGGG
FRO115	start of insert in pRG backbones [4]	modular cloning of all plasmids	forward	TAATACGACTCACTATAGGG
FRO4337	start of insert in pRG backbones [4]	modular cloning of all plasmids	forward	TATATTTCTTTTCGCGGCCG
FRO4342	end of promoter in pRG backbones [4]	modular cloning of all plasmids	reverse	GAATTCCTGCAGCCCGG
FRO4343	P2tet [1]	pCL12	forward	atatacacccagggtctagaAAAGGTTGAAACCAGTTCCCT
FRO4347	start of ORF in pRG backbones [4]	modular cloning of all plasmids	forward	ACTAGTGGATCCCCCGG
FRO4348	end of insert in pRG backbones [4]	modular cloning of all plasmids	reverse	TATCTGTAAATACGGCCGAA
FRO4353	cyc1 terminator, phosphodegron [5] overhang	pCL21	forward	attaaaacgaatgctgcgtcccaggcaaaaactcctttgTAAACGCGTTTAAAGCTTATCCT
FRO4354	phosphodegron [5]	pCL21	forward	acccatctccgcgtccaatgaaaaaaaaaattgagaactttATTAAAACGAATGCTGCGTC
FRO4355	phosphodegron [5]	pCL21	forward	tgagcaaacccatccctagagagtttagtggtttacttacACCCATCTCCGCGTC
FRO4356	phosphodegron [5]	pCL21	forward	ggaagcgattcaaaaaggggcaatatccctaaaccgttaaactTGAGCAAACCCATCCCT
FRO4357	b112 [8]	pCL21	reverse	tattgccccttttgaatcgcttccAAGCTTGAAACACAAATCAGTACCA
FRO4438	P2tet(mut) (see supp. table 3)	pCL33	forward	tcattgaatagtataaaagctattcattgaatagGTAATTCTGTAAATCTATTTCTTAAACT
FRO4439	P2tet(mut) (see supp. table 3)	pCL33	forward	ttgaaaccagttccctgaaattattcccctactatTCATTGAATAGTATAAAAGCTATTC
FRO4440	P2tet(mut) (see supp. table 3)	pCL33	forward	gtggctttgtaaggtatgtgttgctctagaaaaggTTGAAACCAGTTCCCTG

Supplementary Table 5 – Quantitative PCR primers used in this work.

Target	Sequences	Source
lexA	TGGGCTTACTGACCAACCTG<br>CCTGATCATGGAGGGTCAAA	12
tetR	CGCCCAGAAGCTAGGTGTAG<br>TCTCAATGGCTAAGGCGTCG	2
citrine	GGTTGAATTAGATGGTGATGTTA<br>GGCAATTTACCAGTAGTACAAA	8

Supplementary Table 6 – Model fixed parameter values.

Name	Meaning	Fixed value	Reference
ϵ	small number used to avoid numerical errors	10^{-10}	
k_{mat}	citrine maturation rate (min^{-1})	0.0173	[8]
Ch_1	concentration of chemical activating TF_1 added at the same time as α (nM)	5000	as in our experimental setup with β-estradiol
K_{d1}	dissociation constant for Ch_1 binding to TF_1 (nM)	0.1	[8] for β-est
ρ	growth rate of *S. cerevisiae* in YPD (min^{-1})	0.0077	using typical doubling time of 90 min

Supplementary Table 7 – Model optimized parameter specifications.

name	meaning	bounds	explored in log space	projected value (if projectable)
Ch_2	concentration of chemical deactivating TF_2 added at the same time as α (nM)	$[0.110^3]$	yes	0
K_{d2}	dissociation constant for Ch_2 binding to TF_2 (nM)	[0.1100]	yes	10^8
U_{sc}^{nM}	scaling constant: nM per act1 producing unit (nM apu^{-1})	$[10^{-3}\ 10^3]$	yes	1
d_1	TF_1 degradation rate (min^{-1})	$[10^{-4}\ 10^{-1}]$	yes	1
k_{d1}	fold increase of TF_1 degradation rate with phospho-degron (-)	[1 100]	yes	1
k_{L1}	basal production rate of TF1-induced promoters relative to their max production rate (-)	$[10^{-4}\ 0.5]$	yes	1
n_1	Hill coefficient for promoter induction by TF_1 (-)	[0 10]	no	0
d_2	TF_2 degradation rate (min^{-1})	$[10^{-4}\ 10^{-1}]$	yes	1
k_{d2}	fold increase of TF_2 degradation rate with phospho-degron (-)	[1 100]	yes	1
k_{L2}	remaining production rate of TF2-repressed promoters relative to the max production rate (-)	$[10^{-4}\ 0.5]$	yes	1
n_2	Hill coefficient for promoter repression by TF_2 (-)	[0 10]	no	0
$k_{L\alpha}$	basal production rate of α-induced promoters relative to their max production rate (-)	$[10^{-4}\ 0.5]$	yes	
n_α	Hill coefficient for promoter induction by α (-)	[0 10]	no	
$n_{\alpha d}$	Hill coefficient for induced degradation by α with the phospho-degron (-)	[0 10]	no	0
$Km_{\alpha d}$	Km value for induced degradation by α with the phospho-degron (apu)	$[1\ 10^3]$	yes	10^8
**for the following rows: x = α or 1, y = TF1 or TF2.**				
$k_{Px2-ORF}$	max production rate of ORF by the hybrid $\text{P}_{x/2}$ promoter. (apu.min^{-1})	$[10^{-2}\ 100]$	yes	0
$Km_{Px2-ORF}$	Km value for TF_x inducing the production of ORF at the hybrid $\text{P}_{x/2}$ promoter (apu)	$[10^{-1}\ 10^4]$	yes	0
$Km_{P2x-ORF}$	Km value for TF_2 repressing the production of ORF at the hybrid $\text{P}_{x/2}$ promoter (apu)	$[10^{-1}\ 10^4]$	yes	10^8

Supplementary Table 8 – Parameter projection couplings.

$\forall x, \forall y$, **if the following are projected**	**project also**
$k_{Px2-TFy}$	$Km_{P2x-TFy}$ & $Km_{Px2-TFy}$
$Km_{Px2-TFy}$	$k_{Px2-TFy}$
$k_{P12-TFy}$ & $k_{P\alpha 2-TFy}$	d_y k_{dy}, k_{Ly},n_y, $Km_{P12-TFy}$, $Km_{\alpha 2TFy}$,$Km_{P21-TFy}$, $Km_{2\alpha TFy}$
d_y	$k_{P12-TFy}, k_{P\alpha 2-TFy}$
k_{Ly}	$k_{P12-TFy}, k_{P\alpha 2-TFy}$
n_y	$k_{P12-TFy}, k_{P\alpha 2-TFy}$
k_{d1} & k_{d2}	$Km_{\alpha d}, n_{\alpha d}$
$Km_{\alpha d}$	k_{d1}, k_{d2}
$n_{\alpha d}$	k_{d1}, k_{d2}
Ch_2	U_{sc}^{nM},K_{d2}
K_{d2}	Ch_2, U_{sc}^{nM}
U_{sc}^{nM}	K_{d2}, Ch_2

Supplementary References

1. Aslı Azizoğlu, Roger Brent, and Fabian Rudolf. A precisely-titratable, variation-suppressed transcriptional controller to enable genetic discovery. *bioRxiv*, 2019. doi: 10.1101/2019.12.12.874461. URL `https://www.biorxiv.org/content/10.1101/2019.12.12.874461v1`.

2. J. T. Cuperus, R. S. Lo, L. Shumaker, J. Proctor, and S. Fields. A teto toolkit to alter expression of genes in saccharomyces cerevisiae. *ACS Synth Biol*, 4(7):842–52, 2015. ISSN 2161-5063 (Electronic). doi: 10.1021/sb500363y.

3. Jose A Egea, David Henriques, Thomas Cokelaer, Alejandro F Villaverde, Aidan MacNamara, Diana-Patricia Danciu, Julio R Banga, and Julio Saez-Rodriguez. Meigo: an open-source software suite based on metaheuristics for global optimization in systems biology and bioinformatics. *BMC Bioinformatics*, 15(1):1–9, 2014. ISSN 1471-2105. doi: doi:10.1186/1471-2105-15-136.

4. R. Gnügge, T. Liphardt, and F. Rudolf. A shuttle vector series for precise genetic engineering of saccharomyces cerevisiae. *Yeast*, 33(3):83–98, 2016. ISSN 0749-503x. doi: 10.1002/yea.3144. URL `http://dx.doi.org/10.1002/yea.3144`.

5. R. M. Gordley, R. E. Williams, C. J. Bashor, J. E. Toettcher, S. Yan, and W. A. Lim. Engineering dynamical control of cell fate switching using synthetic phospho-regulons. *Proc Natl Acad Sci U S A*, 113(47):13528–13533, 2016. ISSN 0027-8424. doi: 10.1073/pnas.1610973113. URL `http://dx.doi.org/10.1073/pnas.1610973113`.

6. C. Lormeau, M. Rybinski, and J. Stelling. Multi-objective design of synthetic bio-

logical circuits. *Ifac Papersonline*, 50(1):9871–9876, 2017. ISSN 2405-8963. doi: 10.1016/j.ifacol.2017.08.1601. URL <Go to ISI>://WOS:000423965100141.

7. S. Mangan and U. Alon. Structure and function of the feed-forward loop network motif. *Proceedings of the National Academy of Sciences*, 100(21):11980–11985, 2003. doi: 10.1073/pnas.2133841100. URL https://www.pnas.org/content/pnas/100/21/11980.full.pdf.

8. D. S. Ottoz, F. Rudolf, and J. Stelling. Inducible, tightly regulated and growth condition-independent transcription factor in saccharomyces cerevisiae. *Nucleic Acids Res*, 42(17):e130, 2014. ISSN 0305-1048. doi: 10.1093/nar/gku616. URL http://dx.doi.org/10.1093/nar/gku616.

9. Mikołaj Rybiński, Simon Möller, Mikael Sunnåker, Claude Lormeau, and Jörg Stelling. Topofilter: a matlab package for mechanistic model identification in systems biology. *BMC Bioinformatics*, 21(1):1–12, 2020. ISSN 1471-2105. doi: doi:10.1186/s12859-020-3343-y. URL https://bmcbioinformatics.biomedcentral.com/articles/10.1186/s12859-020-

10. M. Sunnaker, E. Zamora-Sillero, R. Dechant, C. Ludwig, A. G. Busetto, A. Wagner, and J. Stelling. Automatic generation of predictive dynamic models reveals nuclear phosphorylation as the key msn2 control mechanism. *Sci Signal*, 6(277): ra41, 2013. ISSN 1937-9145 (Electronic). doi: 10.1126/scisignal.2003621. URL http://www.ncbi.nlm.nih.gov/pubmed/23716718.

11. T. Toni, D. Welch, N. Strelkowa, A. Ipsen, and M. P. H. Stumpf. Approximate bayesian computation scheme for parameter inference and model selection in dynamical systems.

Journal of the Royal Society Interface, 6(31):187–202, 2009. ISSN 1742-5689. doi: 10.1098/rsif.2008.0172. URL `<Go to ISI>://WOS:000262757200006`.

12. T. J. Walton, G. Li, T. A. McCulloch, R. Seth, D. G. Powe, M. C. Bishop, and R. C. Rees. Quantitative rt-pcr analysis of estrogen receptor gene expression in laser microdissected prostate cancer tissue. *Prostate*, 69(8):810–9, 2009. ISSN 0270-4137. doi: 10.1002/pros.20929.

13. E. Zamora-Sillero, M. Hafner, A. Ibig, J. Stelling, and A. Wagner. Efficient characterization of high-dimensional parameter spaces for systems biology. *BMC Syst Biol*, 5:142, 2011. ISSN 1752-0509. doi: 10.1186/1752-0509-5-142. URL `http://dx.doi.org/10.1186/1752-0509-5-142`.
