# Supplementary material for: A rationally engineered decoder of transient intracellular signals
Source: Nat Commun. 2021 Mar 25;12:1886. doi: 10.1038/s41467-021-22190-4 (PMC7994635; doi:10.1038/s41467-021-22190-4)
Supplement: Supplementary file 2 — Reporting Summary [file 41467_2021_22190_MOESM2_ESM.pdf]

nature research

Corresponding author(s): Joerg Stelling

Last updated by author(s): Mar 2, 2021

Reporting Summary

Nature Research wishes to improve the reproducibility of the work that we publish. This form provides structure for consistency and transparency in reporting. For further information on Nature Research policies, see our Editorial Policies and the Editorial Policy Checklist.

Statistics

For all statistical analyses, confirm that the following items are present in the figure legend, table legend, main text, or Methods section.

n/a	Confirmed	
☐	☒	The exact sample size (*n*) for each experimental group/condition, given as a discrete number and unit of measurement
☐	☒	A statement on whether measurements were taken from distinct samples or whether the same sample was measured repeatedly
☒	☐	The statistical test(s) used AND whether they are one- or two-sided<br>*Only common tests should be described solely by name; describe more complex techniques in the Methods section.*
☒	☐	A description of all covariates tested
☒	☐	A description of any assumptions or corrections, such as tests of normality and adjustment for multiple comparisons
☐	☒	A full description of the statistical parameters including central tendency (e.g. means) or other basic estimates (e.g. regression coefficient) AND variation (e.g. standard deviation) or associated estimates of uncertainty (e.g. confidence intervals)
☒	☐	For null hypothesis testing, the test statistic (e.g. *F*, *t*, *r*) with confidence intervals, effect sizes, degrees of freedom and *P* value noted<br>*Give P values as exact values whenever suitable.*
☐	☒	For Bayesian analysis, information on the choice of priors and Markov chain Monte Carlo settings
☒	☐	For hierarchical and complex designs, identification of the appropriate level for tests and full reporting of outcomes
☐	☒	Estimates of effect sizes (e.g. Cohen's *d*, Pearson's *r*), indicating how they were calculated

Our web collection on statistics for biologists contains articles on many of the points above.

Software and code

Policy information about availability of computer code

Data collection	BD FACSDiva 8.1 software was used to collect the flow cytometry data. Roche LightCycler® 96 SW 1.1 software was used to collect the RT-qPCR data.
Data analysis	Matlab R2019a (Mathworks, Natick, MA) was used to analyze the data, and to develop the TopoDesign method described in detail in Supplementary Methods. To analyze FACS data, we used the toolbox MatlabCytofUtilities available from https://github.com/nolanlab/MatlabCytofUtilities. The TopoDesign method depends on the Matlab toolboxes Hyperspace (https://gitlab.com/csb.ethz/HYPERSPACE, commit of 09/17/2018), TopoFilter v0.3.6 (https://git.bsse.ethz.ch/csb/TopoFilter), IQM Tools v1.2.2.2 (https://iqmtools.intiquan.com/) and the 2014 MEIGO-M package (available from http://gingproc.iim.csic.es/meigom.html).<br>All code is available as a static snapshot at the ETH Research Collection with identifier [https://doi.org/10.3929/ethz-b-000471160] and in version-controlled form at https://gitlab.com/csb.ethz/topodesign_decoder.

For manuscripts utilizing custom algorithms or software that are central to the research but not yet described in published literature, software must be made available to editors and reviewers. We strongly encourage code deposition in a community repository (e.g. GitHub). See the Nature Research guidelines for submitting code & software for further information.

Data

Policy information about availability of data

All manuscripts must include a data availability statement. This statement should provide the following information, where applicable:

- Accession codes, unique identifiers, or web links for publicly available datasets
- A list of figures that have associated raw data
- A description of any restrictions on data availability

All computational and experimental data that support the findings of this study are available at the ETH Research Collection with the identifier [https://

doi.org/10.3929/ethz-b-000471160].

Field-specific reporting

Please select the one below that is the best fit for your research. If you are not sure, read the appropriate sections before making your selection.

[x] Life sciences   [ ] Behavioural & social sciences   [ ] Ecological, evolutionary & environmental sciences

For a reference copy of the document with all sections, see nature.com/documents/nr-reporting-summary-flat.pdf

Life sciences study design

All studies must disclose on these points even when the disclosure is negative.

Sample size	For each flow cytometry data point we measured a single aliquot, and about 4000 cells per aliquot after gating to reliably estimate population means and variances. Sample sizes for characterization experiments were chosen to provide sufficient coverage (in terms of model-based identification, see Methods) of (a) dynamics in time-course experiments (n>=5 independent samples) and (b) dose-response curves (n>=8 independent samples). For assessing circuit functionality (Fig. 6c,d), a minimum of three biological variants was used for control and test circuits. qPCR experiments used four technical replicates to allow for estimation of measurement accuracy.
Data exclusions	No data were excluded from the analyses.
Replication	All attempts at replication of the experimental findings in the same conditions were successful. Replication of the findings with high copy number strains that stayed at 4 degrees for some days was not always possible because of loss of gene copies.
Randomization	Samples were not randomized because positioning of samples in multi-well plates (in qPCR experiments) and order of data acquisition by FACS are not expected to affect the conclusions.
Blinding	Blinding was not applied because the automated methods for (model-based) data analysis used are agnostic to sample identities.

Reporting for specific materials, systems and methods

We require information from authors about some types of materials, experimental systems and methods used in many studies. Here, indicate whether each material, system or method listed is relevant to your study. If you are not sure if a list item applies to your research, read the appropriate section before selecting a response.

Materials & experimental systems

n/a	Involved in the study	
[x]	[ ]	Antibodies
[ ]	[x]	Eukaryotic cell lines
[x]	[ ]	Palaeontology and archaeology
[x]	[ ]	Animals and other organisms
[x]	[ ]	Human research participants
[x]	[ ]	Clinical data
[x]	[ ]	Dual use research of concern

Methods

n/a	Involved in the study	
[x]	[ ]	ChIP-seq
[ ]	[x]	Flow cytometry
[x]	[ ]	MRI-based neuroimaging

Eukaryotic cell lines

Policy information about cell lines

Cell line source(s)	All cell lines are derived from FRY69 which comes from the following reference:<br>Gnugge, R., Liphardt, T. & Rudolf, F. A shuttle vector series for precise genetic engineering of Saccharomyces cerevisiae. Yeast 33, 83-98, doi:10.1002/yea.3144 (2016).
Authentication	Cell lines were authenticated by multiplex colony PCR, method published here:<br>Gnugge, R., Liphardt, T. & Rudolf, F. A shuttle vector series for precise genetic engineering of Saccharomyces cerevisiae. Yeast 33, 83-98, doi:10.1002/yea.3144 (2016).
Mycoplasma contamination	The cell lines were not tested for mycoplasma contamination.
Commonly misidentified lines (See ICLAC register)	No commonly misidentified cell lines were used in the study.

Flow Cytometry

Plots

Confirm that:

☒ The axis labels state the marker and fluorochrome used (e.g. CD4-FITC).

☒ The axis scales are clearly visible. Include numbers along axes only for bottom left plot of group (a 'group' is an analysis of identical markers).

☒ All plots are contour plots with outliers or pseudocolor plots.

☒ A numerical value for number of cells or percentage (with statistics) is provided.

Methodology

Sample preparation	Described in detail in the methods section (2 full paragraphs)
Instrument	BD LSR Fortessa cell analyzer equipped with a high-throughput sampler
Software	BD FACSDiva 8.1 software and Matlab R2019a with MatlabCytofUtilities toolbox available at https://github.com/nolanlab/MatlabCytofUtilities.git
Cell population abundance	~ 4000 cells after gating for budding cells in the FSC-W SSC-W plane
Gating strategy	We broadly gated for budded cells in the FSC-SSC-W plane as shown in Extended Data Fig. 10.

☒ Tick this box to confirm that a figure exemplifying the gating strategy is provided in the Supplementary Information.
